# The fluid dynamics and functional diversity of the flagella of free-living flagellates

**DOI:** 10.1098/rsta.2024.0269

**Published:** 2025-09-11

**Authors:** Thomas Kiørboe, Sei Suzuki, Alastair Simpson

**Affiliations:** ^1^ Centre for Ocean Life, DTU Aqua, Technical University of Denmark, Kgs Lyngby, Denmark; ^2^ DTU Aqua, Technical University of Denmark, Lyngby, Hovedstaden, Denmark; ^3^ Department of Biology, Dalhousie University, Halifax, Nova Scotia, Canada

**Keywords:** flagellate foraging, hairy flagella, vaned flagella, fluid dynamics, foraging trade-offs

## Abstract

Flagellates, unicellular organisms equipped with one or a few flagella, are phylogenetically and functionally hugely diverse. Yet, most studies have focused on a few model organisms and on the role of the flagellum in propulsion, ignoring the fundamental role of the flagellum in foraging. The number and position of flagella vary between species; the flagella may be naked or equipped with vanes or hairs; the kinematics and wave patterns vary and may be planar or three-dimensional; and the flagellum may extend from the surface of the cell or lie within a groove on the cell surface. All these features impact the fluid dynamics and functioning of the flagellum. Here we explore some of this functional diversity with a focus on the fluid dynamics of phagotrophic foraging. Finally, we identify gaps in our knowledge of flagellar functioning in this diverse and ecologically significant group of organisms.

This article is part of the theme issue ‘Biological fluid dynamics: emerging directions’.

## 1. Introduction

**Figure 1.**
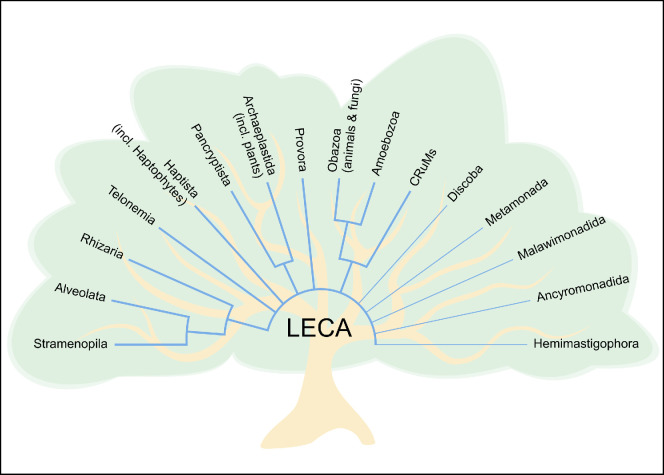
The eukaryotic tree of life. Flagellates are found in all the major branches. Animals, fungi and plants are sub-branches within the Obazoa and Archaeplastida, respectively. Examples of phagotrophic flagellates scattered across the tree are shown in figure 2. Updated from [1].

Flagellates, single-celled organisms equipped with one or a few flagella, are found in all the major branches of the eukaryotic tree of life (figure 1) [2]. They are thus the most diverse assemblage of eukaryotes on this planet. The flagellum (cilium) is key to the functioning of flagellates, and its internal ‘9 + 2’ structure has been conserved throughout evolution. Originally, possibly having evolved with a sensory function [3], the flagellum in contemporary flagellates serves the additional purposes of propulsion, attachment, generation of feeding currents and capture and handling of prey [4,5].

**Figure 2.**
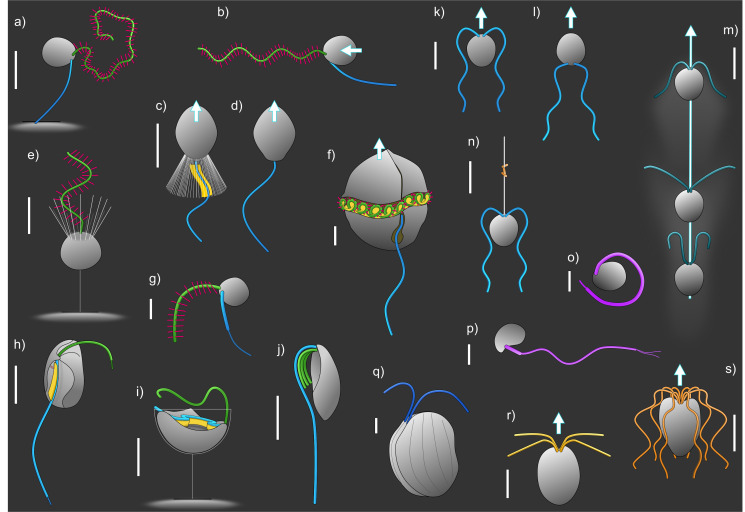
Schematics illustrating the diversity of flagellates scattered across the eukaryotic tree (figure 1). Stramenopiles with three-dimensional (a) or planar waves (b,e); foraging (c) and swarmer stage (d) of choanoflagellate (Obazoa); percolomonad (Discoba) with one long (blue) and three short (green) flagella functioning like a sheet (j); dinoflagellate (Alveolata) (f); colponemid (Alveolata) with vane (yellow on the posterior flagellum) (h); jakobid (Discoba)—a typical excavate with vane (yellow) on the posterior flagellum beating in a ventral groove (i): tiny flagellates of *Minorisa minuta* (Rhizaria) (o); *Micromonas* sp (Archaeplastida) (p); and tiny Stramenopile (g); generalized biflagellate (based on Archaeplastida) with undulatory beat pattern in either normal (k) or reversed (escape) configuration (l); biflagellate (based on Archaeplastida) with ciliary breaststroke beat pattern (m); haptophyte with bacteria (orange) attached to the haptonema (Haptista) (n); *Goniomonas*, a cryptomonad (Pancryptista) (q); and flagellates (Archaeplastida) with four (r) or eight (s) symmetrically organized flagella. Scale bars are 5 µm, except g, o, p (1 µm).

Physicists have a long tradition of studying propulsion of ciliated ‘microswimmers’ at low Reynolds numbers (Re = *UL/ν*, where *U* and *L* are the swimming speed and diameter, respectively, of the swimmer, and *ν* is the kinematic viscosity of the fluid). Despite the phylogenetic and functional diversity of flagellated protists, most physics work has concentrated on a few model organisms, i.e. animal sperm [6], the green alga *Chlamydomonas reinhardtii* [7] and abstract ‘digital twin’ organisms (e.g. the ‘squirmer’) [8]. This has revealed fundamental insights into propulsion mechanisms, including coordination of cilia, steering, sensing and responses to external cues (light, solutes), as well as the structure and biogenesis of cilia [9], and it has stimulated the development of novel experimental and analytical approaches. However, the focus on propulsion in a few model organisms has largely ignored the fundamental role that flagella play in resource acquisition in flagellated protists as well as the huge functional diversity of flagellates found in nature. Also, the questions addressed by the physics community are rarely of an ecological nature. In fact, surprisingly, little is known about the natural history of *C. reinhardtii*, i.e. how it copes with and behaves in its natural environment [9]. Because ‘nothing in biology can be understood except in the light of evolution’ [10], this lack of context makes it difficult to understand how the diverse capabilities of the flagellum and the behaviour of the cell can be understood as adaptations to its survival, resource acquisition and propagation in nature, as well as to the optimization of the trade-offs between these fundamental components of its Darwinian fitness. Organismal trade-offs are the source of diversity and the reason that we find so many fascinating forms of flagellates in nature, the functional ecology of which we will explore here.

As important primary producers and the main consumers of bacteria and picophytoplankton in the ocean, free-living flagellates shape the structure and function of the ocean ‘microbiome’ and thus play a fundamental role in ocean biogeochemistry and climate regulation [11]. There is, therefore, a need to mechanistically understand the functioning of quantitatively important flagellates in their natural environment to better understand and quantify their roles. Free-living flagellates can be photosynthetic (autotrophic) or they can feed on prey (phagotrophic), but many can do both (mixotrophic) [12]. Here we will focus on predatory flagellates and the role of the flagellum in generating a feeding current, from which prey is harvested. Except for dinoflagellates, the abundant phagotrophic flagellates in the water column tend to be very small, typically 1.5−10 µm in linear dimensions [13]. Thus, uptake of dissolved nutrients is mainly governed by diffusion at small Peclet numbers, while encounters with prey particles are impeded by viscosity (Peclet number, Pe = *UL/D*, where *D* is the diffusion coefficient of the solute). Here we review recent work on the diverse ‘mechanistic’ adaptations of flagella and flagellates to propulsion, phagotrophic resource acquisition and survival in their natural environment, as well as outline some of the unsolved problems. We hope to attract the interest of physicists to explore the fascinating diversity of free-living flagellates and to harness the substantial analytical power of the physics community to address problems rooted in ecology rather than in physics itself.

## 2. Morphological and functional diversity of phagotrophic flagellates

The morphological diversity of free-living flagellates is significant (figure 2). The stereotypic description of a flagellate that is pushed by a trailing naked flagellum with near-planar sinusoid waves, as in animal sperm, is rare among free-living flagellates. It is found in the swarmer stage of some choanoflagellates, the closest unicellular relatives of animals (electronic supplementary material, online video 1), as well as in a few other groups or species that have secondarily lost one of the two flagella in the ancestral flagellate. When all flagellates are considered, we find that the number and position of flagella varies markedly (with two, positioned nearer the anterior of the cell, being most common), that the flagella may be naked or equipped with hairs or vanes of different types, that the kinematics and wave patterns of the flagella differ between species and that the flagella of individual cells often differ from each other. The flagella may extend from the cell, or they may be acting closer to the cell body or even lie within grooves on the cell surface. All these features obviously impact the fluid dynamics and functioning of the flagellum. Unsurprisingly, therefore, models of flagellar action based on naked, sperm-like flagella, while providing good predictions of sperm swimming speeds, fail to predict the swimming speeds of free-living flagellates [14]. This variation in kinematics and morphology is to various degrees adaptation, not only—nor even primarily—to propulsion, but also to the many other functions of flagella in free-living flagellates.

## 3. Force production by the flagellum

### (a) The naked flagellum

**Figure 3.**
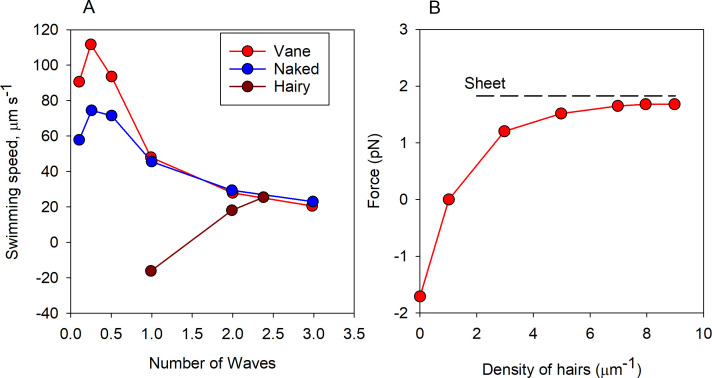
(A). Swimming speed of a hypothetical spherical flagellate with a naked (figure 2d), vaned or hairy (figure 2b) flagellum with a planar beat as a function of the number of waves. The flagellate has a fixed length of the flagellum (10 µm), power consumption (10 fW) and cell size (5 µm), and the flagellum is either pushing (naked, vaned) or pulling the cell. The hairs are oriented in the beat plane of the flagellum, while the vane is oriented perpendicular to the beat plane; note that cells with a trailing vaned flagellum may not exist in nature (redrawn from [16]). (B). Force production of a flagellum equipped with rigid hairs as a function of hair density. The dashed line shows the force production of a sheet with the same wave characteristics as the hairy flagellum. Force direction is towards the cell, and negative force is then away from the cell. Redrawn from [17].

The waving flagellum produces the force necessary for swimming and/or for generating a feeding current. The mechanism of force production by the (near) planar wave of a naked flagellum is well understood and is due to a drag anisotropy [15]: the drag of a filament is larger when it moves sideways than when it moves lengthwise, giving rise to a net thrust. To first order, both the net thrust and the power consumption are proportional to the number of waves, making the thrust per unit power independent of the number of waves. However, as the flagellum becomes increasingly wavy, the thrust units come closer to one another, and hydrodynamic interaction decreases thrust production. Therefore, there is an optimum number of waves to produce the most thrust per unit power invested. Simulations suggest that for cells with a single trailing flagellum (figure 2d), the optimum number of waves is between 0.5 and 1 [16] (figure 3A). This is similar to that found by other optimization procedures [18,19] and to that observed in swimming sperm and in non-feeding swarmer stages of choanoflagellates (electronic supplementary material, online video 1), suggesting that their waveforms are optimized for swimming. Also, a trailing flagellum pushing the cell forward is inefficient for prey encounter since at this scale, viscosity impedes predator–prey contact, and the prey would simply be pushed away by the approaching flagellate [20]

Flagellates with two similar naked flagella may adopt either a breaststroke beat (ciliary beat) or a wavy ‘undulatory’ waveform (figure 2m,k). Both propulsion modes are drag-based, as for the trailing flagellum, but they are both much more efficient in encountering small bacterial prey [16]. Consistent with this, many such cells are mixotrophs [21] even though the best studied such swimmer*—Chlamydomonas*—does not capture prey. Breaststroke swimming is more efficient for propulsion than both the undulatory waveform and the single trailing flagellum for the same power consumption [16,19]. Biflagellated haptophytes with a long haptonema—a long filament used for prey capture (figure 2n)—have long flagella with an undulatory waveform, which turns out to optimize prey capture at the cost of propulsion efficiency [16,22]. Finally, biflagellates with long, wavy flagella may reverse the orientation of the flagella—an escape response—and adopt a highly three-dimensional beat pattern at high frequency to race away from danger (figure 2l) [23]. The beat pattern and consequent fluid dynamics of such escape responses remain to be analysed.

### (b) The hairy flagellum

**Figure 4.**
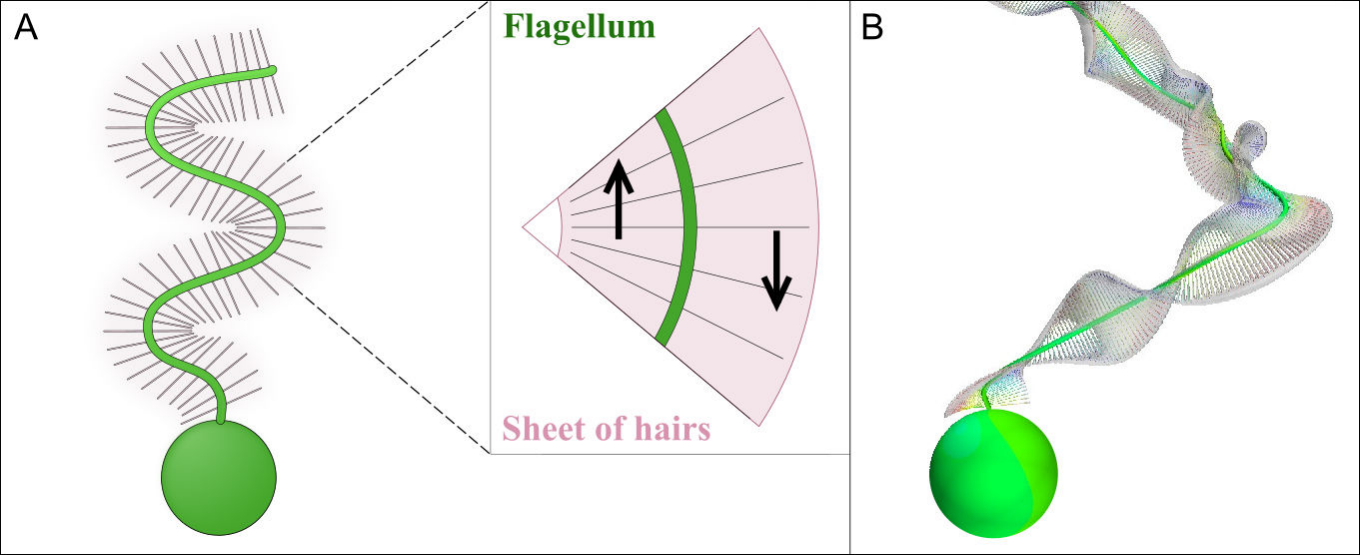
(A) Mechanism of thrust production in a flagellum with a planar beat and equipped with rigid hairs oriented in the beat plane (schematic, not to scale). The hairs function as a flexible sheet. The inserted section of the flagellum shows how the hairs at the crest fan out on the side of the flagellum that moves towards the cell (downward pointing arrow) and come close together on the side that moves away from the cell (upward arrow), thus creating an area-anisotropy that generates net thrust towards the cell. Modified from [17]. (B) Still image of the three-dimensional beat of the flagellum of *Pseudobodo* sp. The hairs form a sheet that is twisted along the length of the flagellum, such that it is oriented near perpendicular to the motion of sections of the flagellum that move towards the flagellum. The idealized spherical cell measures 5 µm.

In many flagellates, the flagella are equipped with hairs. In one of the dominating groups in the ocean, the Stramenopila, the flagella have long (approx. 1 µm) rigid tubular hairs that are different from the ‘softer’ fibrous hairs found in most other hair-bearing flagellates [24]. The soft hairs appear not to affect thrust production [25]. In contrast, the rigid hairs reverse the direction of the force and increase its magnitude by a factor of 5−10 [26,27]. The rigid hairs are oriented in the beat plane of the flagellum in flagella with a planar wave. Thrust reversal of the hairy flagellum has traditionally been explained by a reversal of the drag anisotropy by the individual hairs oriented perpendicular to the flagellum [27,28], thus ignoring hydrodynamic interaction between the closely spaced hairs (typically approx. 7 hairs µm^−1^). However, computational fluid dynamics (CFD) simulations demonstrate that hydrodynamic interaction cannot be ignored and that the densely spaced hairs function as a flexible sheet rather than as individual hairs (figure 3B) [17]. In this case, thrust production is simple and intuitive: net thrust is created at the crests of the waving flagellum, where the hairs fan out on the side of the flagellum that moves towards the cell and come close on the side that moves away from the cell, and this area-anisotropy creates net thrust towards the cell (figure 4A). The net thrust is proportional to the curvature and to the number of waves along the length of the flagellum, consistent with the observation that hairy flagella typically are very curvy and have two or more waves, unlike the naked flagella (compare electronic supplementary material, online videos 1 and 2). The thrust generated by the beating flagellum pulls rather than pushes the cell through the water. However, when feeding, many flagellates, including most Stramenopila, attach to a surface and the waving flagellum therefore generates a feeding current *towards* the cell, and prey arriving in the feeding current are perceived and captured by the flagellum and transported to the cell surface for ingestion (electronic supplementary material, online video 3) [29,30]. The reversal of the feeding current and the consequent flow past the sensing flagellum before arriving at the cell is of obvious adaptive value.

Many Stramenopila with rigid hairs beat their flagellum in a three-dimensional pattern. An extreme example is *Pseudobodo* (figure 2a). When swimming, the hairy flagellum has planar waves that pull the cell through the water (electronic supplementary material, online video 2). When foraging, the flagellate attaches to a surface and winds up its flagellum into a three-dimensional lasso shape, generating a feeding current through the lasso loop towards the cell as waves propagate outwards along the curved flagellum (electronic supplementary material, online video 4). The orientation of the hairs along the length of the flagellum is critical for thrust production. The hairs are permanently attached to the surface of the flagellum with a fixed orientation relative to the central microtubule pair in the flagellum interior [31]. As the flagellum winds up in its three-dimensional pattern, the hair orientation must follow the twist and torsion of the flagellum. The densely spaced hairs function as a sheet, and to yield thrust of the direction and magnitude observed in living cells, CFD simulations suggest that there must be waves of twist-torsion of alternating sign propagating along the beating flagellum, and at the same frequency as the flagellum beats, approximately 50 Hz. This will make the sheet of hairs oriented perpendicular to the local motion of the flagellum when it moves towards the cell and aligned with the motion when it moves away from the cell, generating a net thrust—and a feeding current—through the lasso loop towards the cell (figure 4b) (work in preparation). Recently, such waves of twist and torsion were demonstrated in the flagellum of *Euglena* [32] and visualized in the flagella of *Chlamydomonas* [33], but the amplitude of the twist-torsion in *Pseudobodo* is 60 degrees, compared to the modest 20 degrees estimated for *Chlamydomonas*.

### (c) Flagella with a vane

Many flagellates are equipped with a vane, a wing-like structure that gives the flagellum a ribbon-like appearance. Vanes of various forms are found among dinoflagellates (Alveolata), choanoflagellates (Obazoa), the various excavate groups (Discoba, Metamonada, Malawimonada), as well as in some other forms among Alveolata and Provora (figure 2). In the cases known, the vane is oriented perpendicular to the beat plane, which makes intuitive sense, since the flagellum then may ‘shovel’ more fluid as it beats. However, this effect is rather limited according to CFD simulations (figure 3A) [16]. This can be understood by comparing with the drag force per unit length (*F*_d_) of an infinitely long cylinder at low Re: it increases only a little with the diameter of the cylinder (much less than proportionally) ($F_{d}=4\pi \mu U/log(\frac{7.4\mu }{aU})$, where *U* is the speed, *µ* the kinematic viscosity and *a* the radius of the cylinder [34]).

However, vanes are typically found in flagella that beat in some sort of constriction: in dinoflagellates and excavates, the flagellum beats in a surface groove, while in choanoflagellates, it beats within the space created by the ‘collar filter’ surrounding the flagellum (figure 2c,f,h,i). Choanoflagellates are filter feeders, and the beating flagellum forces water through the collar filter, where bacterial prey is retained. In choanoflagellates, a broad vane on the flagellum was predicted from fluid dynamic considerations: only by postulating a vane was it possible to account for the observed flow through the collar filter and experimentally measured clearance rates in a number of marine species, given estimates of filter resistance (from filter dimensions) and the force generated by a naked flagellum (from beat kinematics) [35]. While a vane had been observed in a few freshwater species, a vane was only visualized in a marine choanoflagellate by advanced Cryo-electron microscopy techniques several years later [36].

While the vaned flagellum operating in the collar filter of choanoflagellates makes the flagellum pump function like a peristaltic pump that squeezes water along, a similar functionality does not apply to vaned flagella that operate in a more open groove, like in excavates and dinoflagellates (figure 2f,h,i). In typical excavate flagellates, prey is harvested from the flow that passes through the groove [37]. In the absence of a groove, the flow generated by a vaned flagellum would create a current with intense sideways flows that cancel out during a complete beat cycle. Operating in a groove, however, yields a more directionally persistent flow and a higher volume pumping (clearance) rate.

Adding a vane to a flagellum operating in a groove is also an energy-efficient way of increasing the pumping rate. Both the power consumption and the pumping rate increase in proportion to the width of the flagellum. Increasing instead the beating frequency similarly increases the pumping rate proportionally. However, at low Re, the power consumption increases with the beat frequency squared, making this an energetically inferior strategy.

### (d) Attached versus free swimming

As noted above, many flagellates attach to a surface when foraging. The cell can either attach directly to the surface, it can attach by a ‘stalk’ raising the cell away from the surface (figure 2i), or it can use one of their flagella to attach, also elevating the cell from the surface. The following two issues are relevant for attached cells: (i) how the proximity of the attachment surface influences the feeding current, and (ii) whether the efficiency of the feeding current, quantified as the clearance rate per unit effort, is different between freely swimming and attached individuals.

The first issue was examined by several authors by comparing observed and modelled flow fields of attached flagellates and other suspension feeders [38,39]. With the feeding flow of the attached flagellate described as a point force working above a no-slip surface and utilizing the analytical solution of Blake [40], a good description of the observed feeding flow is provided [30,39,41]. The presence of an attachment surface reduces the clearance rate. However, depending on the orientation of the force relative to the surface, it reaches 90% of that of an unbounded Stokeslet (point force) if the cell is removed 2 (parallel orientation) or 4 (perpendicular orientation) cell diameters away from the surface. If the feeding current is directed towards the attachment surface, then, in principle, a toroidal eddy with closed streamlines is formed, thus making the cell process the same volume of water repeatedly. However, the recirculation times are so long that the slightest ambient water movement or slight changes in the orientation of the feeding current prevent this [42].

In the ecology literature, it has long been assumed that tethering increases the efficiency of the feeding current [43,44]. This assumption may be based on the shape of the feeding current, which *in a lab frame* is more funnel-shaped and obvious for a tethered than for a freely swimming suspension feeder, but also on a fluid dynamic analysis that ignored the effect of the presence of the cell body [45,46]. Including that effect and watching the flow from the vantage point of the cell reverses the conclusion: a freely swimming suspension feeder will have a higher clearance rate than an attached one, given that the force provided by the flagellum is the same [47]. This conclusion is consistent with computational studies [48].

## 4. Some unexplored and incompletely understood forms

In all the above-mentioned examples, the thrust produced by the flagellum is sufficient to account for observed flow fields, swimming speeds and clearance rates. However, there are multiple morphologies that are incompletely understood and just wait to be explored by biophysicists and functional ecologists. Below are just a few examples:

### (a) Small forms

Very small, 1−2 µm diameter mixotrophic and phagotrophic flagellates abound or even dominate in oligotrophic oceans, and they appear to have very high clearance rates. Most of these species have only been explored through flow cytometry sorting and short-term experiments and live imaging at sea, or through microscopy of preserved cells. Thus, Li *et al.* [49] isolated very small biflagellate haptophytes and measured clearance rates orders of magnitude higher than accounted for by fluid dynamic analyses of larger forms with similar morphology [22]. Small mixotrophic haptophytes appear to be key bacterial grazers in the ocean [21]. Del Campo *et al.* [50] isolated *Minorisa minuta* (figure 2o), a tiny rhizarian flagellate equipped with just one, naked flagellum that appears unhelpful for foraging, yet size-specific clearance rates estimated from their data are similar in magnitude to those of other flagellates. Very small uncultivated stramenopiles, such as certain Marine Stramenopiles, are numerically important phagotrophic flagellates in ocean waters, but most are poorly characterized morphologically. Kammenaya *et al.* [51], however, identified abundant small stramenopiles with a very short hairy flagellum (figure 2g), too short to produce more than half a wave and thus likely functioning differently from stramenopiles with long flagella. The oligotrophic ocean is dominated by small, non-motile bacteria subject to significant Brownian motion, and predator–prey encounter rates in these very small flagellates may, to a significant degree, be accounted for by Brownian diffusion of the bacteria [5], but this cannot account for all the observations. Thus, the mechanisms of propulsion and foraging in the smallest predators in the ocean remain open issues.

Another tiny flagellate, *Micromonas pusilla* (figure 2p), has a single flagellum with an extremely short full axoneme (<1 µm) but with the central pair of microtubules 5 µm long and further extended by ultrathin hairs at the distal end [52,53]. The central pair takes a helical shape and continuously rotates anticlockwise, pushing the cell forward, much like how the bacterial flagellum works. Rotation of the central pair has been found in a few other flagellates, but the mechanism of rotation is unclear. Whether the species is mixotrophic or purely autotrophic is controversial [54,55], but if it does capture prey, the mechanism is entirely unclear. Another rotary motion of a eukaryote flagellum, this time the whole axoneme, is found in the Amoebozoan amoeboflagellate *Idonectes vortex*. It has a single flagellum held in a curve around the cell while rotating, while the cell body rotates more slowly in the opposite direction, generating a fluid flow that resembles a ‘toroidal swimmer’ [56].

### (b) Feeding on other eukaryotes

While most phagotrophic flagellates feed on bacteria that are much smaller than themselves, some flagellates are eukaryovores (i.e. they feed on other eukaryotes, usually of sizes similar to or larger than their own size). This includes an assemblage of distantly related flagellates, which we here call ‘excavate-like flagellates’, that share morphological features with the typical excavates, i.e. a vaned flagellum beating in the ventral groove (figure 2h) [57,58]. However, despite the similarity, according to somewhat anecdotal evidence, they appear to function very differently [57]. In particular, the prey does not fully enter the ventral groove during initial contact, and the organisms do not appear to generate an obvious feeding current. Rather, the predator seems simply to swim directly into the prey. The encounter between equal-sized particles is much less impeded by viscosity than when a big predator approaches a much smaller prey, but the involved mechanisms remain to be explored and understood. The same applies to many telenomid flagellates [59]. Most species appear not to produce a feeding current. For example, some species have two short trailing flagella (e.g. *Telonema subtilis*) that push the cell forward, and they can swim directly up to the prey. Others have one lateral hairy flagellum and a smooth trailing flagellum, but the fluid dynamics of propulsion is unclear. They may attach with the smooth flagellum and make circular movements while beating with the hairy one (https://disk.yandex.ru/i/CNUO8MANlmZntQ). It has been speculated that this generates an incoming flow—a feeding current—from which prey is harvested [59], but the details of prey capture, the fluid dynamics and the exact role of the flagellum are unknown.

More detailed descriptions and fluid dynamic analyses exist for dinoflagellates, which are also eukaryovorous (figure 2f). They create a feeding current by the activity of a flagellum that runs in a groove around the circumference of the cell. This flagellum is embedded in a ‘sock’, thus forming a waving sheet (vane), and is further equipped with hairs of unknown orientation and rigidity [60]. The waving flagellum propels the cell and creates a feeding current that is directed towards the prey capture area [61]. It also makes the cell rotate, however, in the same direction as the direction of the travelling wave. This oddity can be reproduced in CFD simulations [62], but the mechanism is not well understood. It may well be that it involves dynamic twist-torsion of the flagellum and the sheet, as has been described in the flagellum of *Euglena* that—like dinoflagellates—have a paraxonemal rod running along the axoneme [32].

## 5. Conflicting roles of the flagellum: trade-offs

As noted above, the flagella of free-living flagellates often serve multiple purposes, e.g. both propulsion and foraging, but morphology, wave kinematics, etc. may not simultaneously optimize both, leading to potential design trade-offs. Also, generating a feeding current creates a fluid disturbance that may be perceived by flow-sensing predators, leading to a consequent foraging-risk trade-off. Such organismal trade-offs are subject to natural selection, and flagellates may be differently adapted to different environments.

### (a) The near-field flow

Michelin & Lauga [63] used a squirmer model to explore the foraging-swimming trade-off and found that the squirmer configuration that maximizes swimming efficiency is also the configuration that maximizes feeding rate. They considered food a diffusing passive scalar. That description applies to the uptake of dissolved nutrients in autotrophic flagellates, but it is also a good approximation to the consumption of small (approx. 0.5 µm diameter) non-motile bacteria subject to Brownian motion. However, it does not work for foraging on larger bacteria or on motile bacteria because these typically have average run lengths much longer than the size of the flagellate, where the diffusion analogy breaks down.

Also, while the squirmer model may be a fair model of ciliates with a near even surface distribution of many cilia, like *Paramecium* (another model organism), it is a poor model for most planktivorous ciliates that have elaborate ciliary arrangements that are highly specialized for feeding current generation and capture of food [64], as well as for most flagellates that have only a few flagella. Many flagellates have two flagella—or multiples of two (4, 8, 16; figure 2r,s)—arranged symmetrically, and the flagella typically have one or both of two distinct wave patterns: a breaststroke ciliary beat with power and recovery strokes that leads to a puller arrangement, or a more continuous undulatory beat that leads to either a pusher or a puller arrangement depending on the orientation of the flagella (figure 2k,l). Models varying in complexity, as well as observed flow fields, all agree that the ciliary beat optimizes swimming, while the undulatory flagellar beat optimizes prey encounter [16,19,22]. The undulatory mode also minimizes the hydrodynamic ‘noise’ and, hence, exposure to flow-sensing predators. Similarly, in flagellates with a hairy flagellum, having many waves optimizes swimming and stealth but is suboptimal for foraging [16]. Thus, there are clear trade-offs.

### (b) Architecture of the far-field flow

While the near-field flow field generated by the flagella and considered above depends on details in the flagellar wave pattern and morphology, the architecture of the far-field flow is often well described by very simple point-force models that ignore most of the morphological details illustrated above [26,30,65,66]. It depends mainly on the number and position of flagella and on whether the flagellate is attached or freely swimming. Thus, a swimming flagellate produces a flow field that is often well approximated by a stresslet, i.e. two oppositely directed point forces of equal magnitude, while the flow field produced by an attached flagellate is well approximated by a Stokeslet, i.e. a force working in a point. The presence of the attachment surface, of course, modifies the flow depending on the distance to the surface and the orientation of the force; however, in both cases, analytical solutions exist that resemble observed flows [30,39,40,42].

The Stokeslet and the stresslet produce flows that attenuate with one over distance and one over distance squared, respectively. Thus, everything else being equal, an attached flagellate generates a flow that can be perceived by a predator further away than a freely swimming one. However, a ‘breaststroke’ swimmer, such as *Chlamydomonas* and many other biflagellates, produces an even more rapidly attenuating flow field, as one over distance cubed. It can be described by a three-point Stokeslet organized on a line (quadrupole), where two Stokeslets represent the forces produced by the two flagella, and a third Stokeslet between the two represents the oppositely directed force from the moving cell body [67]. Mapping the far-field flow from many different flagellates and estimating both the efficiency of the feeding current and the extension of the fluid disturbance reveal a clear foraging-risk trade-off: the most efficient foragers are also those that run the highest risk of being detected by a flow-sensing predator [26].

## 6. Conclusions

The phylogenetic and functional diversity of phagotrophic flagellates is huge. While some species are known only from their molecular signature, microscopy-based morphological descriptions exist for many species, and new species are continually being discovered. However, only for a subset of the many functional types do we have descriptions of swimming and foraging behaviour, and in even fewer of these, an understanding of the underlying fluid mechanics and the implied organismal trade-offs, e.g. between foraging efficiency and predator avoidance.

Marine microbial communities play a key role in ocean biogeochemistry, and the structure and function of these communities are principally governed by organismal trade-offs and environmental conditions [68]. Trait-based approaches to model marine microbial communities utilize exactly this, and the reliability and predictive capability of such models is much improved if based on mechanistically underpinned—rather than heuristically derived—functional ecology and organismal trade-offs [69].

Progress in mechanistically understanding the functional ecology of important phagotrophic flagellates is impeded by the difficulty of keeping many species in culture. Yet, existing culture collections, both institutional and those held by individual research groups, still contain many species to be explored. Such endeavours have a considerable scope for the discovery of novel biological solutions to challenges of moving, feeding and avoiding being fed upon, and hopefully, this overview will inspire biophysicists to engage in examining this diversity.

## Data Availability

Supplementary material is available from [70].
